# Mechanistic studies of MALAT1 in respiratory diseases

**DOI:** 10.3389/fmolb.2022.1031861

**Published:** 2022-11-07

**Authors:** Wenzheng Wu, Shihao Wang, Lu Zhang, Beibei Mao, Bin Wang, Xiaoxu Wang, Dongsheng Zhao, Pan Zhao, Yunying Mou, Peizheng Yan

**Affiliations:** ^1^ College of Pharmacy, Shandong University of Traditional Chinese Medicine, Jinan, China; ^2^ College of Chinese Medicine, Shandong University of Traditional Chinese Medicine, Jinan, China; ^3^ The Second Affiliated Hospital of Shandong University of Traditional Chinese Medicine, Jinan, China

**Keywords:** long-stranded non-coding RNA, lung cancer, metastasis-associated lung adenocarcinoma transcript, respiratory disease, severe acute respiratory syndrome coronavirus

## Abstract

**Background:** The incidence of respiratory diseases and the respiratory disease mortality rate have increased in recent years. Recent studies have shown that long non-coding RNA (lncRNA) MALAT1 is involved in various respiratory diseases. In vascular endothelial and cancer cells, MALAT1 expression triggers various changes such as proinflammatory cytokine expression, cancer cell proliferation and metastasis, and increased endothelial cell permeability.

**Methods:** In this review, we performed a relative concentration index (RCI) analysis of the lncRNA database to assess differences in MALAT1 expression in different cell lines and at different locations in the same cell, and summarize the molecular mechanisms of MALAT1 in the pathophysiology of respiratory diseases and its potential therapeutic application in these conditions.

**Results:** MALAT1 plays an important regulatory role in lncRNA with a wide range of effects in respiratory diseases. The available evidence shows that MALAT1 plays an important role in the regulation of multiple respiratory diseases.

**Conclusion:** MALAT1 is an important regulatory biomarker for respiratory disease. Targeting the regulation MALAT1 could have important applications for the future treatment of respiratory diseases.

## Introduction

Respiratory diseases are widespread, and are characterized by primary pathology of the trachea, bronchi, lungs and chest. They manifest as coughing, chest pain, obstructed breathing, and respiratory failure. Respiratory diseases are the third most common cause of death in urban areas and the leading cause of death in rural areas, and air pollution is a major contributor (Li et al., 2021). The morbidity and mortality from respiratory diseases is increasing owing to the aging of the population, unhealthy lifestyles, and worsening atmospheric pollution. Therefore, it is important to study the pathogenesis of respiratory diseases and to develop targeted drug therapies for these diseases.

Long non-coding RNAs (lncRNAs) are a group of non-coding RNA transcripts with a length of >200 nucleotides. Although knowledge of their role is limited, lncRNAs are thought to perform a number of regulatory functions (Najafi, 2022). LncRNAs function as a signal, decoy, guide, scaffold, and microRNA (miRNA) modulator in affecting biological processes and preserving homeostasis (Rinn and Chang, 2012; Mirzaei et al., 2022a), and are involved in gene expression in various pathologies (Qu et al., 2019) (Table 1).

**TABLE 1:** Pathophysiological expression of long non-coding RNA.

Area of action	Physiological effects
Gene	Transcription in upstream promoter region of gene interferes with downstream gene expression. MALAT1 blocks the association of EZH2 with HIV5-LTR and reduces the epigenetic modification of LTR by PRC2
Gene	Inhibits RNA Pol II or mediates chromatin remodeling and histone modification; affects downstream gene expression. MALAT1 can promote HIV-1 replication by antagonizing EZH2-mediated transcriptional silencing of viral genes
Gene	Forms complementary double strands with gene transcripts, interferes with mRNA shearing, and forms different types of shearing. Tripathi et al. (2010) confirmed that nuclear-enriched MALAT1 interacts with serine-arginine (SR) proteins by RNA-FISH experiments using MALAT1 and U2 snRNA-specific probes and antibodies to SR splicing factors, affecting, among other things, the distribution of splicing factors
Gene	Forms complementary double strands with gene transcripts and produces endogenous siRNA. LncRNAs can target and regulate multiple carcinogenic pathways by binding to form siRNA.
Protein	Binds specific proteins and regulates activity of corresponding proteins. Overexpression of MALAT1 causes increased production of inflammatory proteins
Protein	Forms nucleic acid-protein complexes as structural components. During pneumonia, MALAT1 binds to the NF-κB signaling pathway and forms a complex
Protein	Binds specific proteins and alters their cellular localization. Blocking lncRNA MALAT1 regulates EMT and prevents tumor metastasis
microRNA	Precursor molecule for miRNA or piRNA. The identification of lncRNAs as precursors of miRNAs has been corroborated by BLASTN.

Abbreviations: EMT, epithelial–mesenchymal transition; EZH2, Enhancer of zeste homolog 2; FISH, fluorescence *in situ* hybridization; HIV, human immunodeficiency virus; lncRNA, long non-coding RNA; LTR, long terminal repeat; miRNA, microRNA; NF-κB, nuclear factor kappa-light-chain-enhancer of activated B cells; piRNA, PIWI-interacting RNA; Pol II, polymerase II; PRC2, polycomb repressive complex 2; siRNA, short interfering RNA; snRNA, small nuclear RNA; SR, serine-arginine.

The lncRNA MALAT1 is closely associated with the development and progression of respiratory diseases (Zhang et al., 2021). This review provides the first comprehensive analysis of the molecular mechanisms by which MALAT1 regulates the pathophysiology of various respiratory diseases and clarifies its relevance to complications of these diseases. It highlights its potential therapeutic role and provides a source of reference for future research on related diseases and screening of candidate drugs.

## MALAT1 overview

The lncRNA MALAT1 is a non-coding RNA that commonly occurs in malignant tumor cells. The MALAT1 gene is encoded on human chromosome 11q13.1 and mouse chromosome 19qA, and is located in gene-dense regions with very high synthetic evolutionary conservation (Djebali et al., 2012).

**FIGURE 1:**
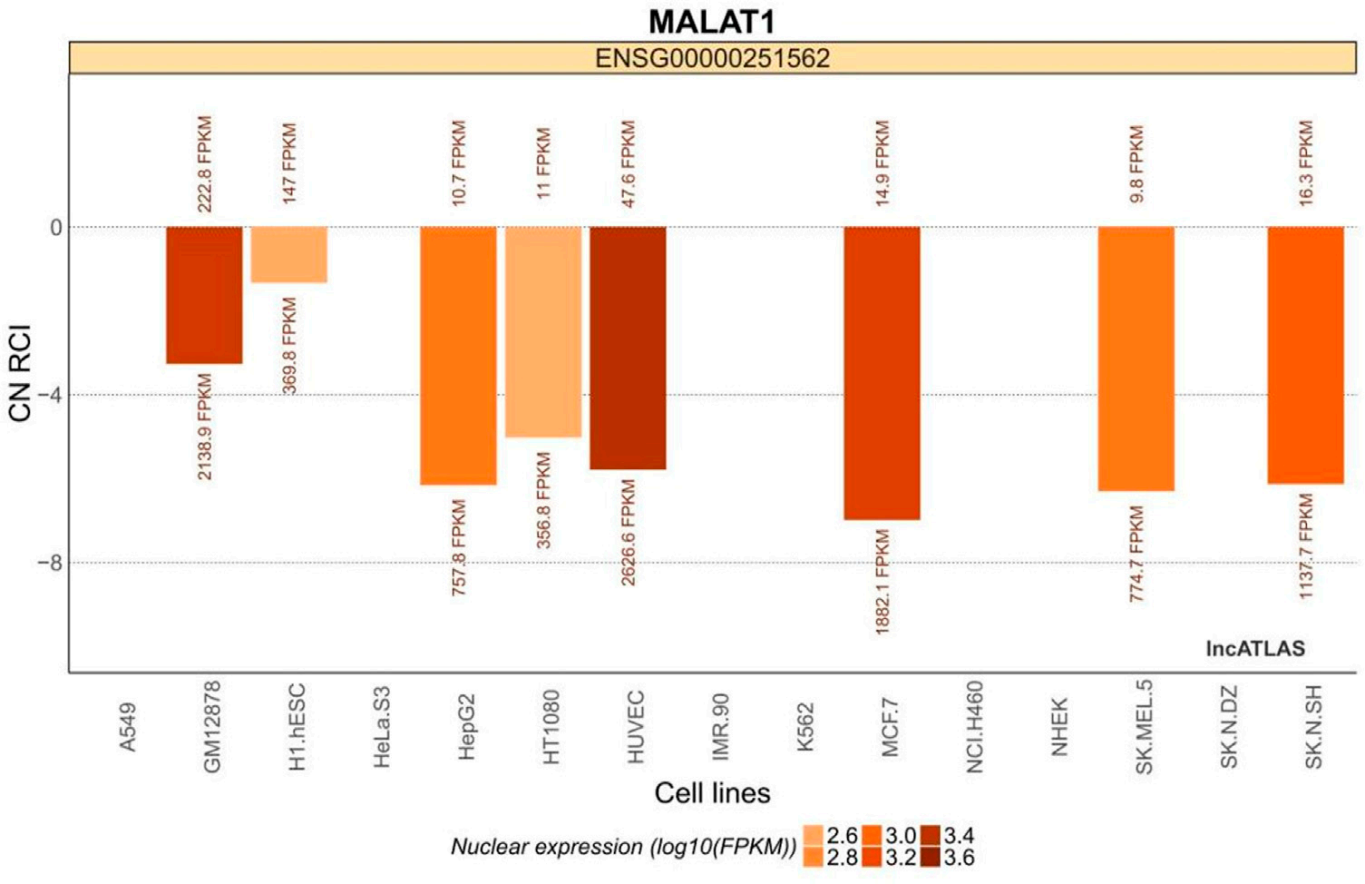
MALAT1 expression in common cell lines. The relative concentration index (RCI) was calculated using RNA-Seq data for each cellular fraction harvested by ensemble. The values above and below each cluster bar graph are the number of reads expressed by MALAT1 in the cytoplasm and nucleus of that cell line, respectively. The CN RCI values for MALAT1 were expressed in both the cytoplasm and the nuclei but were relatively more abundant in the latter. Positive values indicate localization to the cytoplasm and negative values indicate localization to the nucleus. Absolute values increase with localization intensity. Abbreviations: C, cytoplasmic; N = nuclear.

MALAT1 is expressed in a variety of common cell lines (Figure 1). It is expressed in both the cytoplasm and the nucleus, but is more abundant in the nucleus (Mas-Ponte et al., 2017).

Data on the relative concentration index (RCI) were used to assess the potential roles of MALAT1. The RCI was calculated according to the following formula:

Where RPKM = reads per kilobase transcript mapped per million mapped reads, i = the number of reads of MALAT1 in the cytoplasm of the cell line, and j = the number of reads of MALAT1 in the cytoplasm of the cell line.

**FIGURE 2:**
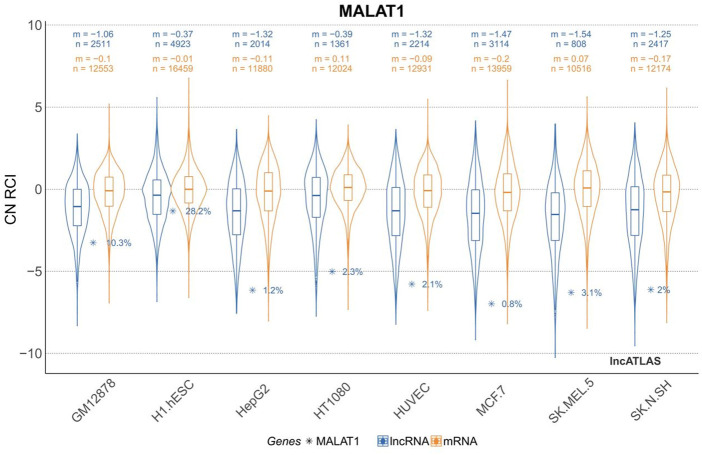
Cytoplasmic-to-nuclear relative concentration index (RCI) of MALAT1 in different cell lines. m, median RCI value per group; n, number of genes per group.

**FIGURE 3:**
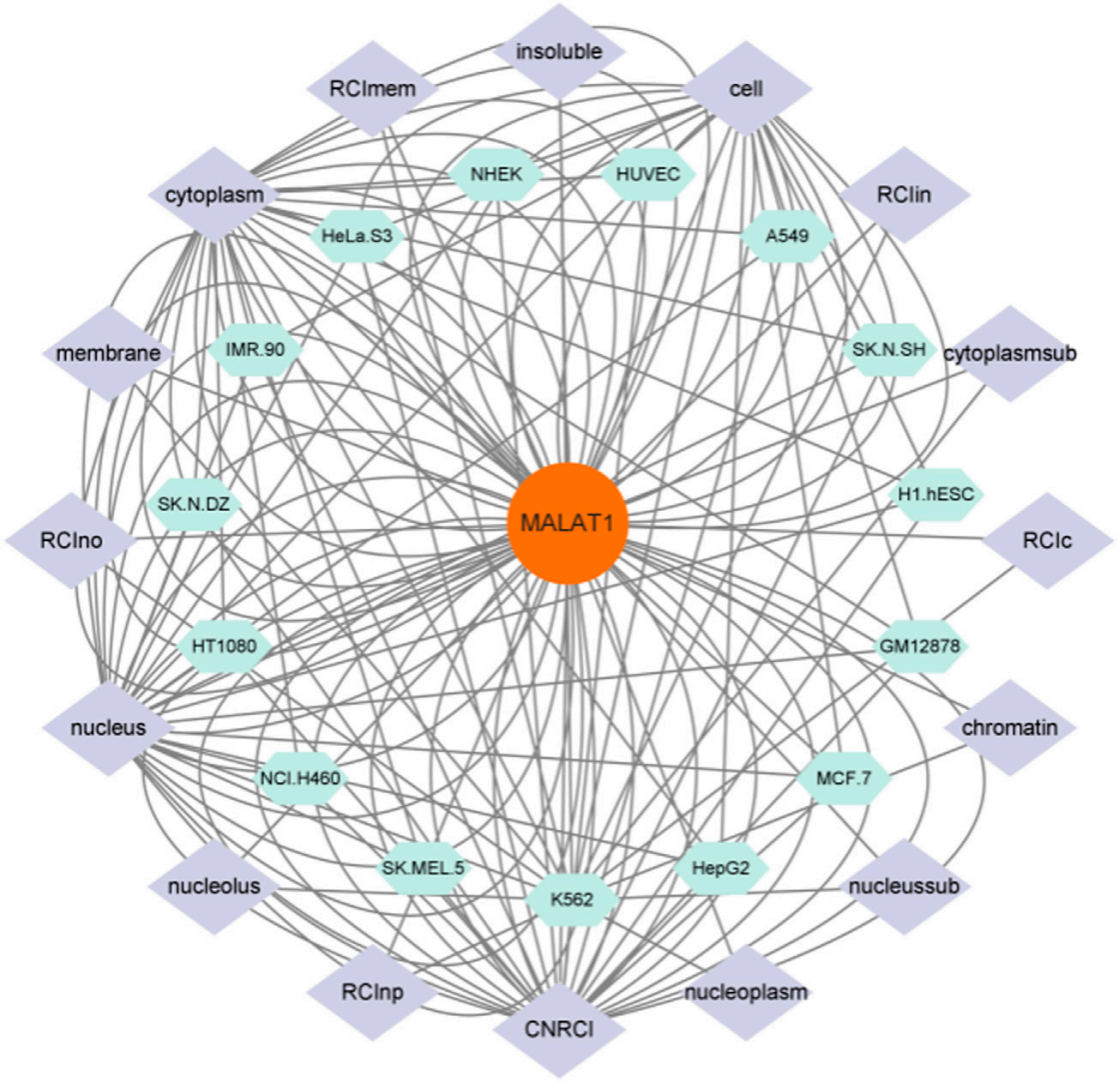
Figure 3 shows the cell lines in which MALAT1 expression is present and the targets of action of these cell lines, and is a visual analysis of Figures 1, 2. As shown in the figure the green section shows the cell line on which MALAT1 acts, and the purple section shows the specific location of the action.

The H1.hESC and GM12878 cell lines had cytoplasmic-to-nuclear (CN) RCI of 28.2% and 10.3%, respectively, showing that MALAT1 is actively expressed in embryonic cells and B lymphocytes, and therefore might play a role in cancer cell proliferation, differentiation, migration, and humoral immunity. These findings provide a theoretical basis for mechanistic studies on the association between MALAT1 and specific respiratory diseases. MALAT1 can act on multiple cell targets within different cell lines. Thus, it exerts various physiological effects. Tables 2 and Figure 3 were generated using the cell lines in Figure 2 with MALAT1 present and the proportion of cells with MALAT1 therein.

**TABLE 2:** Presence of MALAT1 in various cell lines.

Cell line	Name of human cell line	Proportion of cells with MALAT1 (%)
H1.hESC	Embryonic stem	28.2
GM12878	B lymphocytes	10.3
SK.MEL.5	Melanoma	3.1
HT1080	Fibrosarcoma	2.3
HUVEC	Umbilical vein vascular endothelial	2.1
SK.N.SH	Neuroblastoma	2.0
HepG2	Liver cancer	1.2
MCF.7	Breast cancer	0.8

As shown in Figure 3, MALAT1 can be expressed at multiple targets in various cell lines, which suggests that MALAT1 can act at different sites to produce different pathophysiological responses. Finally, we summarize in Table 3.

**TABLE 3:** MALAT1-mediated micro RNA regulatory networks in respiratory diseases.

Literature source	Sponged miRNAs	Target genes/signaling pathways	Biological functions
Tang et al.	miR-206	PI3K/Akt	Promotion of lung cancer proliferation and metastasis
Gutschner et al.; Arun et al.	Endogenous microRNA	PI3K/Akt, Wnt/β⁃catenin	Regulation of lung cancer cell proliferation, invasion, metastasis, and apoptosis
Zhuo et al.	miR-214	--	Inhibition of vascular endothelial cell proliferation *in vitro*
Liang et al.	miR-155	T-bet	Reduction of Th1/Th2 ratio
Yang et al.	miR-129-5p	--	Enhancement of cell viability and interruption of BPD development
Yan et al.	miR-503	PI3K/Akt/mTOR/Snail	Promotion of silica-induced pulmonary fibrosis development and progression
Liu et al.	miR125b, miR-146a, miR-203	MAPK/NF-κB	Induction of tissue and lung damage

Abbreviations: BPD, bronchopulmonary dysplasia; MAPK, mitogen-activated protein kinase; miR, microRNA; miRNA, microRNA; mTOR, mammalian target of rapamycin; NF-κB, nuclear factor kappa-light-chain-enhancer of activated B cells; Th, T helper cell.

## MALAT1 and respiratory disease pathophysiology

**FIGURE 4:**
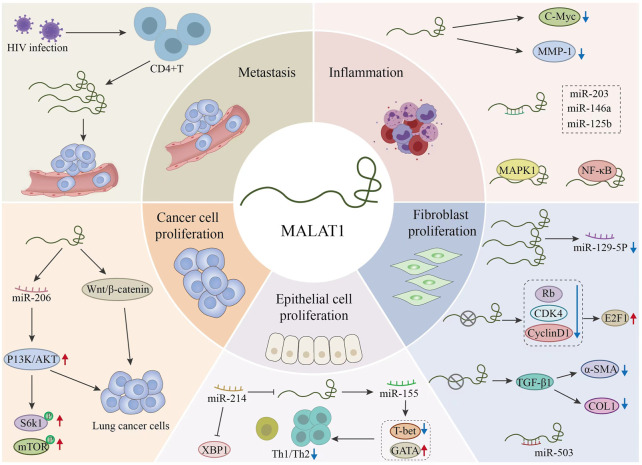
Regulation of different microRNAs (miRNAs) by MALAT1 and the related targets of action of MALAT1.This figure explains the pathophysiology related aspects in the process of related respiratory diseases through MALAT1. MALAT1 further affects the development of the disease by regulating related signal pathways, proteins and miRNAs.

MALAT1 was first identified in non-small cell lung cancer (NSCLC). In the early stages of disease, MALAT1 can be used to identify patients at high risk of metastasis. It is also an important indicator of tumor prognosis. Additionally, MALAT1 regulates various pathophysiological processes such as proliferation, invasion, metastasis, and apoptosis by targeting the expression of multiple miRNAs and activating different signaling pathways (Luo and Niu, 2022). We plot the regulation results as Figure 4.

### Proliferation

#### Cancer cell proliferation

Cell proliferation is the increase in the number of cells caused by cell division (cytoplasmic division), and it is the final step in the cell cycle. It is necessary for normal tissue development and maintenance during the life cycle. Cell proliferation is a tightly regulated process, and many different proteins control cell cycle checkpoints. Genetic mutations found in cancer cells can lead to uncontrolled cell proliferation.

MiRNAs are important for transcriptional regulation. MALAT1 acts as a molecular sponge and is a highly efficient inhibitor of miRNAs. MALAT1 is equivalent in potency to antisense oligonucleotides and can be regulated by sponge adsorption. It thus inhibits the proliferation of lung cancer cells and slows disease progression.

Cancer development is initiated when a single mutant cell proliferates. Genetic mutations lead to the creation and rapid growth of malignant tumors (Cooper et al., 2000). MALAT1 promotes lung cancer cell proliferation by targeting and regulating miR-206 expression, activating the PI3K/Akt signaling pathway, and increasing phosphorylation of mammalian rapamycin (mTOR), ribosomal protein S6 kinase 1 (S6K1) and other related proteins (Tang et al., 2018). MALAT1 competes with endogenous miRNAs, regulates the expression levels of downstream genes and controls the proliferation, invasion, metastasis and apoptosis of lung cancer cells through various signaling pathways such as PI3K/Akt and Wnt/β-catenin. Specific antisense oligonucleotide treatment against MALAT1 inhibits tumor growth in mouse breast cancer and human NSCLC metastasis models (Arun et al., 2016; Gutschner et al., 2013). Thus, MALAT1 can regulate the miR-206-induced PI3K/Akt signaling pathway and promote cancer cell proliferation through sponge adsorption. This suggests that MALAT1 may be an important target for cancer therapy. The potential role of MALAT1 in cancer therapy could be explored by conducting MALAT1 inhibitor experiments in mice followed by the simultaneous application of cancer impact factors in a control group.

#### Vascular endothelial cell proliferation

Angiogenesis is the biological process whereby pre-existing blood vessels form new blood vessels. It is a critical process that promotes development, skeletal muscle hypertrophy, menstruation, pregnancy, and wound healing. Angiogenesis can also lead to pathological conditions, such as neovascular disease (e.g., retinopathy), rheumatoid arthritis, psoriasis, AIDS-related Kaposi’s sarcoma, and cancer (tumorigenesis). Angiogenesis is a complex, highly ordered process that relies on an extensive network of signaling between and within endothelial cells, their associated wall cells (vascular smooth muscle cells and pericytes), and other cell types (e.g., lymphocytes).

Pathological and physiological angiogenesis share many similarities in terms of signaling activities and changes in cellular function and behavior; therefore, angiogenesis has potential therapeutic applications. However, one of the key differences is that pathological angiogenesis does not terminate when tissue perfusion is inadequate. This uncontrolled, haphazard, and ill-defined growth can prevent the development of new interrupting angiogenic mediators (Chung et al., 2010).

The rs619586A>G single nucleotide polymorphism of MALAT1 inhibits vascular endothelial cells *in vitro* by acting as a competing endogenous RNA (ceRNA) for miR-214, thereby upregulating XBP1 and shortening the proliferative phase of S-M changes during the cell cycle (Zhuo et al., 2017). MALAT1 also directly or indirectly downregulates miR-155, which in turn upregulates GATA-binding protein 3 (GATA-3), downregulates T-bet, and decreases the Th1/Th2 ratio. Thus, MALAT1 expression and the Th1/Th2 ratio are negatively correlated (Liang and Tang, 2020).

#### Fibroblast proliferation

Lung injury may be caused by a variety of factors. In lung injury, the interstitium secretes collagen to effect tissue repair. If alveolar myofibroblasts proliferate and differentiate abnormally and accumulate in the extracellular matrix, lung disease and developmental defects may occur, and may promote the development of bronchopulmonary dysplasia (BPD), chronic obstructive pulmonary disease (COPD), and idiopathic pulmonary fibrosis (IPF).

MALAT1 is a key gene involved in the regulation of lung fibrosis (Wang, 2020). MALAT1 knockdown inhibits myofibroblast formation through epithelial–mesenchymal transition (EMT), downregulates phosphorylated Rb genes, cyclin D1, and CDK4, and causes E2F1 transcription factor overexpression. Hence, MALAT1 positively regulates E2F1 expression through the cyclin D1-CDK4-Rb signaling axis, exerts EMT effects, and promotes the development of pulmonary fibrosis. TGF-β acts as a “master switch,” inducing pulmonary fibrosis and myofibroblast differentiation. MALAT1 silencing decreases *a*-SMA and COL1 mRNA and protein expression in TGF-β1-activated human lung fibroblasts, thus inhibiting their proliferation (Chang, 2019). MALAT1 overexpression downregulates miR-129-5p, promotes cell viability, and blocks the development of BPD. MALAT1 upregulation and miR-129-5p inhibition reduce apoptosis in lung epithelial cells. Nevertheless, persistent dysregulation may exacerbate the inflammatory response and contribute to BPD pathophysiology by upregulating HMGB1 (Yang et al., 2020). Upregulated MALAT1 acts as a ceRNA, and directly binds and downregulates its miR-503 target *via* the PI3K/Akt/mTOR/Snail signaling pathway. This mechanism exacerbates lesion severity and distribution *in vivo* and leads to the development and progression of silica-induced pulmonary fibrosis (Yan et al., 2017). These findings suggest that MALAT1 affects the expression of various miRNAs and signaling pathways and leads to inflammation and pulmonary fibrosis.

### Transfer

Metastasis is the process by which *in situ* cancer cells enter circulation and colonize a new environment. This process is closely associated with respiratory cancer progression. A 1,000-fold knockdown of MALAT1 in human lung cancer cells *via* zinc finger nuclease technology impairs cancer cell migration (Coros et al., 2008; Gutschner et al., 2013), and blocking the lncRNA MALAT1 pathway regulates EMT and combats tumor metastasis (Masuzawa and Kitazawa, 2021).

The results of the preceding studies suggest that MALAT1 is a metastatic biomarker for lung cancer and has the potential to be used therapeutically to prevent metastasis. The potential therapeutic role of MALAT1 in preventing metastasis could be further evaluated by analyzing lung pathology sections and relevant pathophysiological indices through the administration of MALAT1 inhibitors to mice followed by the simultaneous application of cancer-affecting factors to a control group.

### Induction of inflammation

Inflammation is a host defense mechanism that detects and removes harmful exogenous substances and initiates self-healing. Inflammation may be acute or chronic. Acute inflammation usually occurs in response to host invasion by microorganisms and toxins or because of other physical trauma. Chronic inflammation slowly progresses over a long period of time (months to years) and its persistence may not be perceived by the host.

MALAT1 downregulation inhibits the nuclear translocation of *ß*-catenin and reduces c-Myc and MMP-7 levels (Wang et al., 2016). A recent study confirmed the role of MALAT1 in lipopolysaccharide-induced inflammatory responses and suggests that it controls inflammation and natural immunity (Zhao et al., 2016).

MALAT1 is intrinsically correlated with its targets miR125b, miR-146a, and miR-203. Hence, it is also positively correlated with the GOLD stage and proinflammatory cytokines in stable COPD patients. It also interacts with miR-125b, miR-146a, and/or miR-203 to promote inflammation and increase disease severity in patients with COPD. MALAT1 might cause tissue damage and lung injury by regulating its downstream MAPK/NF-κB pathway and exacerbating COPD (Liu S. et al., 2020).

The foregoing experimental findings indicate that MALAT1 aggravates inflammation and tissue damage which, in turn, increases the inflammatory response in the host. Hence, it could serve as a novel drug target for the treatment of various inflammatory diseases. The potential role of MALAT1 in the treatment of inflammatory diseases could be further evaluated by analyzing lung pathology sections and the associated pathophysiological indices through the simultaneous application of inflammatory impact factors to mice following the use of MALAT1 inhibitors with control groups.

## Respiratory diseases

### MALAT1 and COVID-19 pneumonia

Coronavirus disease (COVID-19) pneumonia, caused by severe acute respiratory syndrome coronavirus type 2 (SARS-CoV-2), has been spreading globally since December 2019 and is currently pandemic. SARS-CoV-2 infection can cause high expression of MALAT1, which in turn can cause organ damage. SARS-CoV-2 is highly infectious and its first pass is diffusion through the airway epithelium. It is transmitted to the respiratory tract *via* droplets and primarily affects the respiratory system (Wu et al., 2020; Zhou et al., 2020; Zhu et al., 2020).

Comparison of molecular studies on SARS-CoV and SARS-CoV-2 revealed that they have overlapping pathogenesis. The viral envelope spike (S) protein trimer binds human angiotensin-converting enzyme 2 (ACE2), and human transmembrane serine protease 2 (TMPRSS2) binds the S protein (Li et al., 2003; Hoffmann et al., 2020). Organ-specific clinical manifestations and complications are associated with cellular ACE2 and TMPRSS2 expression (Bertram et al., 2012; Du et al., 2020). The S protein of SARS-CoV-2 binds human ACE2 with 10-fold–20-fold greater affinity than that of SARS-CoV (Wrapp et al., 2020).

During SARS-CoV-2 infection, MALAT1 is highly expressed, affecting cytokine function, airway epithelial cell permeability, and inflammation (Wrapp et al., 2020; Manna et al., 2022). SARS-CoV-2 infection targets airway epithelial cells that hydrolyze angiotensin I (Ang I) into angiotensin II (Ang II), a potent vasoconstrictor (Manna et al., 2022). ACE2 then cleaves Ang II into Ang (1–7) which downregulates the proinflammatory cytokines TNF-α and IL-6 (Qi, 2011). When SARS-CoV-2 binds to ACE2, it decreases Ang II levels, causing inflammation by upregulating proinflammatory factors.

Previous studies have shown a time-dependent increase in viral load (VL). (Devadoss et al., 2021). It has been shown that an increase in the SARS-CoV-2 VL may upregulate MALAT1 which, in turn, may control lncRNAs LASI, TOSL, and NEAT1. LASI, TOSL, and NEAT1 are significantly upregulated in patients with high VLs without a corresponding change in the level of MALAT1 expression (Devadoss et al., 2021). Thus, the correlation between SARS-CoV-2 VL and MALAT1 expression is variable.

SARS-CoV-2 VL is positively correlated with MALAT1 expression. Interference with MALAT1 might restore the normal physiological function of ACE2 and alleviate inflammation. This mechanism could be exploited in the treatment of COVID-19 pneumonia. This could be further evaluated by administering SARS-CoV-2 to mice after the concurrent use of MALAT1 inhibitors in a control group.

Many SARS-CoV-2-positive nasopharyngeal swabs exhibit significant lncRNA NEAT1 and MALAT1 upregulation. In SARS-CoV-2-positive saliva samples, however, only lncRNA NEAT1 is upregulated. In contrast, MALAT1 is highly upregulated in SARS-CoV-2-positive nasopharyngeal samples (Rodrigues et al., 2021), suggesting that either NEAT1 or MALAT1 could be used for SARS-CoV-2 detection and diagnosis.

### MALAT1 and bronchial disease

#### Bronchial asthma

Bronchial asthma is one of the most common chronic lung diseases worldwide. Its prevalence has been increasing as global industrialization advances. It is generally accepted that bronchial asthma is an allergic disease triggered by multiple factors. According to the World Health Organization, asthma affects more than 300 million people worldwide. Delayed treatment may increase mortality (Bousquet et al., 2010; Bateman. et al., 2018).

The pulmonary vasculature is closely associated with the development of asthma. It participates in intrapulmonary gas exchange, the maintenance of homeostasis in the internal environment, and the control of changes in airway airflow. Alterations in bronchial vascular permeability, dilatability, and density during asthma attacks are positively correlated with the severity of the attack, and contribute to the persistence of the disease (Avdalovic, 2015). MALAT1 promotes vascular endothelial cell dysfunction and impedes endothelial remodeling (Michalik et al., 2014). It may also regulate various pathological vascular remodeling processes (Michalik et al., 2014; Brock et al., 2017).

MALAT1 can affect bronchial asthma by regulating vascular endothelial growth factor (VEGF) and Th1/Th2 cell ratios. VEGF is highly expressed in the airways of patients with asthma. It enhances microvascular permeability, induces endothelial cell proliferation, and promotes vascular regeneration. VEGF is an important factor influencing inflammation, airway and vascular remodeling, and associated pathophysiological changes in asthma. VEGF promotes antigen sensitization and a Th2-type inflammatory immune response, increases the number of dendritic cells and activates them, links intrinsic and adaptive Th2-type immune responses, and exacerbates asthma airway inflammation (Lee et al., 2004). Hypoxia-inducing factor (HIF) exists as the subunits HIF-1α/2α and HIF-1β. In hypoxia, HIF-1 binds hypoxic reaction elements in the promoter region of its target genes by translocating to the nucleus and activating gene transcription. During inflammation, the hypoxic microenvironment and/or non-oxygen-dependent mechanisms upregulate HIF-1α. HIF-1 upregulates VEGF in response to hypoxia (Guzman et al., 2008) and aggravates asthma symptoms.

The expression of MALAT1 is negatively correlated with lung function and the Th1/Th2 ratio (Liang and Tang, 2020). It has been hypothesized the MALAT1 could affect miR-155, which could then affect GATA-3, thereby lowering Th1/Th2 ratios and downregulating T-bet. T-bet and GATA-3 regulation by MALAT1 may affect lymphocyte development and, by extension, control bronchial asthma (Linch et al., 2016). T-bet plays an important role in innate lymphoid cell type 3 (ILC3) (Sciumé et al., 2012), and fights infection and maintains epithelial integrity by expressing IL-17 and IL-22 (Sugimoto et al., 2008; Van Maele et al., 2014). GATA-3 promotes the development of ILC2, which produces the Th2 cytokines IL-5, IL-6, and IL-13 (Moro et al., 2010). Innate lymphocytes may be involved in a variety of health-promoting biological processes, including fighting infection (Krishack et al., 2019) and promoting tissue repair (Maeda et al., 2011), but abnormal innate lymphocyte activation may result in asthma (Mjosberg and Spits, 2012), autoimmune diseases (Neumann et al., 2017), and cancers (Kirchberger et al., 2013).

Previous studies suggest that Th2-type hypersensitivity response links MALAT1 to VEGF and builds a regulatory network. VEGF upregulation significantly elevates Th2-type hypersensitivity in patients with asthma. By contrast, MALAT1 is negatively correlated with lung function and the Th1/Th2 ratio. Hence, it induces asthma by regulating and abnormally activating innate lymphoid cells.

MALAT1 inhibitors are currently available for the treatment of certain cancers, but have not been used for the treatment of asthma. The potential role of MALAT1 inhibitors could be evaluated by conducting experiments in mice using MALAT1 inhibitors followed by simultaneous application of induction factors in a control group.

#### Bronchopulmonary dysplasia

BPD is an important cause of respiratory disease in preterm infants, and the incidence is increasing (Collins et al., 2017). However, the survival rate of BPD has improved significantly owing to the widespread use of maternal glucocorticoids and alveolar surfactant replacement therapy. Certain treatments for BPD have a limited effect in preterm infants and the pathogenesis of pediatric BPD remains unknown. BPD is characterized by severe alveolar and pulmonary vascular dysplasia (Hwang and Rehan, 2018). MALAT1 has shown promise in controlling BPD by regulating cell proliferation and apoptosis during lung organogenesis.

MALAT1 is significantly upregulated in the lung tissue of BPD mice. MALAT1 is differentially regulated in the peripheral blood cells of patients with BPD compared with those of normal subjects (Cai et al., 2017). Epigenetic research has shown that DNA methylation plays a regulatory role in respiratory diseases such as BPD. Runt-associated transcription factor 3 (RUNX3) is a gene class associated with lung development, epithelial differentiation, and pulmonary vascular development. After transcription, RUNX3 is regulated through DNA methyltransferase-1- and DNA methyltransferase-3b-mediated DNA methylation. DNA methylation and H3K27me3 epigenetic modifications have been identified in the RUNX3 promoter region in a hyperoxia-induced neonatal mouse BPD model (Zhu et al., 2015), suggesting that DNA methylation plays a regulatory role in BPD.

BPD is associated with oxidative stress. Kelch-like ECH-associated protein 1/nuclear factor red lineage 2-associated factor 2 (KEAP1/NRF2) signaling pathway plays a crucial role in the cellular antioxidant response by defending against various types of external damage, and is considered the most important endogenous antioxidant signaling pathway *in vivo* (Jeong et al., 2006). MALAT1 is significantly downregulated, whereas KEAP1 and NRF2 are significantly upregulated in hyperoxia-treated mice, which exposed to a 92% (v/v) O2/5% (v/v) CO_2_ gas mixture at a rate of 3 L/min for 10 min, suggesting that oxidative stimulation may lead to high flux expression of the KEAP1/NRF2 signaling pathway and significant MALAT1 reduction (Zhang et al., 2021).

In BPD, MALAT1 mediates the KEAP1/NRF2 signaling pathway, thereby diminishing cellular antioxidant capacity and augmenting pathology. DNA methylation may also aggravate BPD. MALAT1 downregulation reflects BPD remission. Nevertheless, it remains to be established whether MALAT1 is linked to DNA methylation and whether it modulates other physiological functions in response to BPD therapy. DNA methylation as one of the pathogenic factors affecting BPD has potential clinical applications.

### MALAT1 and the lung

Endothelial cell damage is an important cause of lung disease. MALAT1 and miR-320a are endothelial dysfunction markers. The MALAT1/miR-320a axis regulates endothelial dysfunction and plays a vital role in endothelial cell reconstitution (Zhao et al., 2021). Furthermore, MALAT1 is an important target of aberrant innate lymphoid cell (ILC) activation. ILC3 modulation could potentially be used to treat lung disease (Van Maele et al., 2014).

#### Pneumonia

MALAT1 regulates the inflammatory response by modulating the nuclear factor-κB (NF-κB) signaling pathway (Lei et al., 2019). Interleukin-8 (IL-8) is a potent chemotactic substance and neutrophil activator associated with the development and maintenance of inflammation. In humans, the airway epithelium is the main source of IL-8, and pneumonia pathogenesis is positively correlated with IL-8 expression. NF-κB participates in MALAT1-mediated regulation of the inflammatory response to *Mycoplasma pneumoniae* infection (Gu et al., 2020). *In vivo* experiments have shown that MALAT1 downregulation reduces lung inflammation caused by *M. pneumoniae* infection.

Moreover, MALAT1 controls VEGF which, in turn, regulates various inflammatory conditions. MALAT1 also affects airway epithelial cells and regulates the NF-κB signaling pathway, which is implicated in several critical physiological functions. The potential role of MALAT1 in the prevention and treatment of lung inflammation could be further evaluated by conducting experiments in mice using MALAT1 inhibitors followed by the simultaneous application of inflammatory impact factors in a control group.

#### Acute lung injury and acute respiratory distress syndrome

Acute lung injury (ALI)/acute respiratory distress syndrome (ARDS) is a severe respiratory disorder with a variety of causes, which is associated with high morbidity and mortality (Mason et al., 2017). The CD14-TLR4-NF-κB signaling pathway is closely related to human inflammatory and immune responses (Ness et al., 2017). Upregulating the CD14-TLR4-NF-κB signaling pathway exacerbated disease progression in a rat ALI/ARDS model. Upregulating the CD14-TLR4-NF-κB signaling pathway increased the expression of lipopolysaccharide-like factors and accelerated the development and progression of ALI/ARDS in rats (Ness et al., 2017).

MALAT1 was one of the first lncRNAs to be associated with human disease, and it plays an important role in lung disease. MALAT1 influences the progression of lung disease by regulating a range of disease-associated molecules. MALAT1 also regulates the suppression of immune responses to tumors and other pathological conditions by controlling MDSC-mediated lung cancer suppression (Zhou et al., 2018). In patients with ALI/ARDS, miR-181a-5p is regulated by MALAT1, and may participate in the inhibition of Fas and apoptosis (Moro et al., 2010). The activation of Fas signaling plays a crucial pathophysiological role in ALI/ARDS-related inflammation and apoptosis (Perl et al., 2007; Mizuta et al., 2008; Messer et al., 2013). Consequently, MALAT1 is an important putative target in ALI therapy.

ALI/ARDS is characterized by sustained and excessive lung inflammation which increases pulmonary capillary permeability and leads to pulmonary edema, hypoxemia, apoptosis, and lung destruction (Fan and Fan, 2018; Herrero et al., 2018). Various miRNAs have different effects on human lung disease (Alipoor et al., 2014). MiRNA-181d is downregulated in the bronchial epithelial cells of smokers (Schembri et al., 2009), and miRNA-181a regulates the inflammatory response of human fibroblasts (Galicia et al., 2014; Qu et al., 2017).

MALAT1 can further influence ALI/ARDS by affecting the CD14-TLR4-NF-κB signaling pathway. This could be further evaluated by performing miRNA assays in mice treated with MALAT1 inhibitors, followed by simultaneous application of the effector in a control group.

Clinical studies have shown that upregulation of the CD14-TLR4-NF-κB signaling pathway accelerates ALI/ARDS development and progression. MALAT1 is upregulated and miR-181a-5p is downregulated in the sera of patients with ALI/ARDS. The relationship between the CD14-TLR4-NF-κB axis and MALAT1 is unclear. However, MALAT1 is thought to exacerbate ALI/ARD progression by regulating physiological functions and the CD14-TLR4-NF-κB axis. Thus, future research into the control of ALI/ARDS should focus on axial regulation. The CD14-TLR4-NF-κB axis appears to be important for the regulation of ALI/ARDS. This could be further evaluated by experiments in mice using impact factors.

#### Lung cancer

Lung cancer is a common malignant tumor of the respiratory system. It has high clinical morbidity and annually increasing mortality rates (Dang et al., 2021). At present, surgical resection is the only clinically effective method of curing lung cancer. However, surgery is not possible in some patients, and chemotherapy and radiotherapy have limited effectiveness in controlling lung cancer. Therefore, effective targets for lung cancer chemotherapy are required.

Lung cancer risk factors include smoking, air pollution, and a family history of the disease. In patients with a family history of lung cancer, women are at a higher relative risk of the disease than men. A prospective study in Japan found that for women and men with a positive history of lung cancer among first-degree relations, the relative risks were 2.65 and 1.69 in women and men. (Nitadori et al., 2006).

Iron death, which is characterized by massive LPO accumulation, is inhibited in lung cancer cells. Hence, inhibition of lipid synthesis also inhibits iron death in lung cancer. Lung cancer cells counteract iron death by regulating lipid metabolism *via* lymphoid-specific helicase, which modifies DNA methylation by inducing genes involved in lipid metabolism, including glucose transporter protein type 1 (GLUT-1), stearoyl-CoA desaturase 1 (SCD1), and fatty acid desaturase 2 (FAD2). These enzymes interact with the WD40 protein family member, WDR76, to inhibit iron death (Jiang et al., 2017). Therefore, iron death modulation could potentially induce and maintain remission in patients with lung cancer. Iron death, as a carcinogenic mechanism, could have important implications for cancer treatment This could be further evaluated by administering cancer impact factors to mice and analyzing the relevant protein pathways regulating iron death.

MALAT1 plays a regulatory role in various types of lung cancer. MALAT1 overexpression inhibits the tumor suppressor PSF (Garen and Song, 2008), and may therefore accelerate tumor growth. MALAT1 is abnormally overexpressed in NSCLC tumor cells and tissues (Schmidt et al., 2011). Myeloid-derived suppressor cells (MDSCs) have immunosuppressive functions and suppress the immune response in tumor and other diseases. Zhou et al. (2018) and Perl et al. (2007) showed that MALAT1 plays a direct role in regulating MDSC expansion. Thus, MALAT1 is a putative drug target for the treatment of several different types of lung cancer. The potential role of MALAT1 in the treatment of lung cancer could be further evaluated by conducting experiments in mice using MALAT1 inhibitors followed by the simultaneous application of cancer impact factors in a control group.

Research to date has not yet linked MALAT1 to iron death. Nevertheless, both modalities can inhibit lung cancer to varying degrees. The lncRNA SNHG1 delays Alzheimer’s disease by regulating iron death (Liu, 2021). Investigating the mechanism of the interaction between MALAT1 and iron death could lead to the development of effective new therapies for various cancers. The proteins that affect iron death could be further evaluated by administering cancer impact factors to mice following the use of MALAT1 inhibitors simultaneously in controls.

An exosome is a minute structure that participates in the regulation of biological process through its contents, which can be a protein, lipid or nucleic acid. Genetic tools and antineoplastic agents may also be inserted into exosomes. Exosomes play a role in intercellular communication and play an essential role in cancer progression or inhibition (Paskeh et al., 2022). Administering MALAT1 in exosomes has a potential role in treating cancer. This is a potential new direction for future drug research and development.

#### Chronic obstructive pulmonary disease

COPD is a heterogeneous disorder characterized by partially reversible persistent airflow limitation, progressive deterioration in lung function, and increased airway obstruction (Diao et al., 2016; Maddocks et al., 2017). The inflammatory responses to transient receptor potential anchor protein subtype 1 (TRPA1) and transient receptor potential vanilloid subtype 1 (TRPV1) mediate interleukin (IL) release. Exogenous stimuli induce an inflammatory response that activates TRPA1 mediating the release of IL-8 and also induce TRPV1, mediating the release of the proinflammatory factor IL-1β. The release of IL-8 induces the inflammatory response (Zhang and Wang, 2017), which is relatively upregulated in patients with COPD. The transient receptor potential cation channel, TRPA1/TRPV1, regulates the excitability of pulmonary sensory neurons in COPD development. TRPA1 and TRPV1 interact with each other and participate in the airway inflammatory response by regulating IL-8 and IL-18 expression (Liu Y. et al., 2020). Current evidence suggests that the airway epithelium is a major source of IL-8 and that MALAT1 regulates airway epithelial cells and mediates IL-8 secretion. By contrast, the mechanisms by which MALAT1 regulates TRPA1 and TRPV1 have not yet been elucidated. This could be further evaluated by administering induction factors to mice and simultaneously administering MALAT1 inhibitors to controls, and by analyzing TRPA1 and TRPV1 level to determine whether they are correlated with overall pathological changes.

COPD is a severe inflammatory lung disease characterized by excessive inflammatory response, inappropriate immune and macrophage activation, and lung injury. The lncRNA MALAT1 is implicated in all these phenomena (Tuder and Petrache, 2012; Dai et al., 2018; Cui et al., 2019). Patients with AECOPD were compared against those with stable COPD and it was discovered that the MALAT1 levels were higher in the former than the latter. Moreover, the MALAT1 levels were substantially higher in patients with AECOPD than normal subjects. MALAT1 also demonstrated clinical relevance in the prediction and management of AECOPD as it is intrinsically correlated with its miR125b, miR-146a, and miR-203 targets (Liu S. et al., 2020). Another study found that MALAT1 expression was relatively lower in patients with COPD with MALAT1 hypermethylation.(Liu Y. et al., 2020). Dual luciferase reporter assays illustrated that miR-146a-targeted MALAT1 and MALAT1 overexpression significantly increased MALAT1 and COX2 mRNA and protein expression which, in turn, downregulated miR146a. Hence, the MALAT1 hypermethylation promoter is associated with mild COPD, improved lung function, and COX2 and PGE1 upregulation (Sun L. et al., 2021). Current evidence suggests that MALAT1 has potential as a novel biomarker for the prediction of COPD severity, and could play a role in the clinical diagnosis and effective treatment of COPD. This could be further evaluated by administering COPD pro-influencing factors to mice and administering MALAT1 inhibitors to a control group.

#### Pulmonary fibrosis

Pulmonary fibrosis comprises numerous end-stage alterations in lung disease including fibroblast proliferation, massive extracellular matrix accumulation, and inflammatory damage and structural destruction of lung tissues. Most cases of pulmonary fibrosis are idiopathic. Although oral pirfenidone and nintedanib impede the progression of pulmonary fibrosis, they have limited effect on the mortality rate. Hence, innovative treatment modalities are required for pulmonary fibrosis (Selvaggio and Noble, 2012; Hughes et al., 2016).

It has been empirically demonstrated that lncRNA prostate cancer-associated transcript 29 (PCAT29) inhibits the miRNA-221-mediated RASAL1/ERK1/2 signaling pathway downstream of transforming growth factor β1 (TGF-β1) in lung fibroblasts, thus suppressing certain proinflammatory cytokines associated with lung fibrosis, such as N4bp2 and Plxna4, and ultimately slowing the progression of pulmonary fibrosis (Liu et al., 2018).

In EMT, adjacent polar epithelial cells are transformed into nonpolar mesenchymal cells lacking intercellular connections and having increased mobility (Wynn and Ramalingam, 2012). EMT plays an important role in the development of lung fibrosis. Experiments revealed that MALAT1 was significantly upregulated, and miR-503 was significantly downregulated, in HBE and A549 cells subjected to silica. Thus, MALAT1 and miR-503 expression are negatively correlated (Yan et al., 2017). Moreover, MALAT1 is upregulated in pulmonary fibrosis caused by silica exposure. Future research should assess the effectiveness of MALAT1 suppression in the treatment of silicosis. This could be further evaluated by applying induction factors to mice and simultaneously applying MALAT1 inhibitors to a control group.

## MALAT1 in medical applications

### MALAT1 in lung disease diagnosis

MALAT1 mediates the regulation of multiple pathophysiological responses and exerts various effects on pulmonary diseases by controlling NF-κB, CD, miR, and cytokines. These mechanisms suggest that MALAT1 could be used to treat different pulmonary diseases. MALAT1 suppresses lung cancer by regulating MDSCs which, in turn, modulate immune responses that inhibit tumors and other diseases. MALAT1 directly regulates MDSC expansion (Perl et al., 2007) and enhances lung adenocarcinoma cell motility (Tano et al., 2010). Blood MALAT1 levels are lower in patients with lung cancer than in healthy controls (Guo et al., 2015). MALAT1 expression has been detected in both *in situ* lung carcinoma and metastatic lymph node tissues, but its levels are higher in lung carcinoma than in lymph node metastases (Fenwick et al., 2015). As MALAT1 can be detected in whole blood, it can be readily used to identify and facilitate the treatment of lung cancer.

In patients with ALI/ARDS, MALAT1 regulates miR-181a-5p and the latter inhibits Fas and apoptosis (Zhou et al., 2018). Fas signaling activation plays important pathophysiological roles in ALI/ARDS-associated inflammation and apoptosis (Perl et al., 2007; Mizuta et al., 2008; Messer et al., 2013). Hence, MALAT1 is an important potential target for ALI treatment.

Currently, bronchodilators and anti-inflammatory drugs administered by inhalation are the mainstay of clinical treatment for COPD. Currently available drugs modulate COPD by reducing lung inflammation. Inhalation of the pan-JAK inhibitor PF1367550 blocks the release of CXCL9, CXCL10, and CXCL11 from BEAS-2B and airway epithelial cells (Fenwick et al., 2015). EGFR mediates mucus hypersecretion and IL-8 expression in airway epithelial cells. The EGFR inhibitor AG-1478 inhibits cigarette smoke-induced mucin synthesis *in vitro* and *in vivo* (Hegab et al., 2007). MALAT1 exerts its effects through its targets miR125b and miR-146a. MiR125b is implicated in numerous responses and also participates in the development of certain diseases (Hui et al., 2018; Shen et al., 2020). MiR125b overexpression is negatively associated with survival in patients with NSCLC and is positively correlated with COPD progression. By contrast, MALAT1 overexpression is negatively correlated with miR-146a expression. MALAT1 is a predictive biomarker of COPD severity, and could be used to treat inflammation by regulating miR. Hence, novel drugs targeting COPD are urgently required.

Targeted therapies for IPF include nintedanib and pirfenidone. Nintedanib mitigates IPF progression by inhibiting platelet-derived growth factor receptor (PDGFR), vascular endothelial growth factor receptor (VEGFR), and fibroblast growth factor receptor (FGFR). The kinases targeted include FGFR1, FGFR2, FGFR3, PDGFR-α, and PDGFR-β. Simultaneous targeting these proangiogenic receptors may enhance their effectiveness as antitumor agents and overcome resistance to VEGF- and VEGFR-2-targeting drugs (Liu et al., 2014; Wollin et al., 2015). In contrast, MALAT1 affects VEGF by modulating Th2-type immune responses. Thus, it has an impact on patients with IPF. Pirfenidone is a potent cytokine inhibitor that impedes fibroblast activity and reduces cell proliferation and stromal collagen biosynthesis by modulating and/or inhibiting certain factors. It reduces lipid peroxidation and the secretion of proinflammatory mediators and has antifibrotic, anti-inflammatory, and antioxidant effects (Feng et al., 2015; Ito et al., 2019). MALAT1 affects cellular inflammation and is a potential reference for targeted drug studies investigating the combination of pirfenidone and MALAT1 in the treatment of IPF. The antifibrotic mechanisms of pirfenidone and nintedanib provide a physiological basis for mitigating the loss of lung function in patients with IPF (Skandamis et al., 2019; Li and Ye, 2020; Song et al., 2020).

### MALAT1 in the diagnosis of bronchial diseases

Though clinical treatments are available for bronchial asthma and BPD, these conditions are nonetheless incurable. Common clinical treatments for bronchial asthma include glucocorticoids, β2-and leukotriene receptor antagonists, and anticholinergic agents. (Han and Wang, 2021). MALAT1 expression is negatively correlated with lung function and the Th1/Th2 ratio, and it affects GATA-3. GATA-3 induces the development of ILC2 which promotes the biosynthesis of the TH2 cytokines IL-5, IL-6, and IL-13. These substances play important roles in the pathophysiology and clinical diagnosis of bronchial asthma. There are few therapeutic interventions available for BPD. They consist mainly treatments of glucocorticoids, mechanical ventilation, and other treatment modalities (Wang and Cheng, 2019) that attenuate bronchospasm, reduce inflammation, and alleviate other symptoms. In airway inflammation, MALAT1 either modulates the NF-κB signaling pathway or reduces the secretion of proinflammatory cytokines. Clarification of the molecular mechanisms of MALAT1 in the BPD process could facilitate the development of targeted drugs.

Clinical studies on MALAT1 can be conducted by using MALAT1 inhibitors or by genetically engineering MALAT1-knockout mice to form experimental groups, and by intervening in the control and experimental groups with relevant causative factors, and testing the affected population to determine whether a direct relationship can be established by measuring MALAT1 levels.

## Conclusion

MALAT1 has an impact on different diseases by directly regulating related signaling pathways or indirectly affecting related protein expression. In COVID-19 pneumonia, MALAT1 modulates the permeability of airway epithelial cell membranes and the normal intracellular physiological function of ACE2 to alleviate inflammation. In bronchial asthma, MALAT1 co-regulates Th2-type hypersensitivity with VEGF, abnormally activates ILC, and causes asthma. MALAT1 mediates the KEAP1/NRF2 signaling pathway, reduces cellular antioxidant capacity, and enhances the pathology of BPD. In pneumonia, MALAT1 modulates the inflammatory response by regulating the NF-κB signaling pathway. The regulatory interaction between MALAT1 and the CD14-TLR4-NF-κB axis can mitigate ALI/ARDS. In lung cancer, MALAT1 overexpression inhibits the tumor suppressor PSF and regulates the amplification of tumor-suppressing MDSCs. MALAT1 affects lung cancer cell proliferation and metastasis through a dual pathway. In COPD, MALAT1 targets miR125b, miR-146a, and miR-203. MALAT1 downregulation alleviates disease symptoms. In pulmonary fibrosis, there is a negative correlation between MALAT1 and miR-503 expression that can inhibit pulmonary fibrosis. Thus, MALAT1 downregulation could also ameliorate this condition.

LncRNAs regulate enhancer of zeste homolog 2 (EZH2) *via* spongy miRNAs. Circular RNAs also regulate the expression of EZH2 signaling in a similar manner to lncRNAs by regulating EZH2 expression *via* targeting miRNAs. EZH2 generally acts as a tumor promoter; however, owing to its methyltransferase activity, it may also act as a tumor suppressor. Among the ncRNAs, miRNAs and lncRNAs play an important regulatory role in EZH2 signaling (Mirzaei et al., 2022b). Thus, investigating whether MALAT1 can influence cancer progression through its regulation of EZH2 warrants further research.

As miRNA sponges, circular RNAs affect downstream genes by suppressing miRNA-associated repression, leading to alterations in the malignant phenotype of cervical cancer cells (Najafi et al., 2022). MALAT1 expression in tissues and secretions (e.g., cervical vaginal lavage) is highly variable and has potential use as a biomarker of cervical cancer, which could be used for clinical diagnosis and treatment (Liao et al., 2020). In patients with ovarian cancer, high levels of MALAT1 expression in localized lesions or in serum exosomes may indicate a poor prognosis (Lin et al., 2018; Qiu et al., 2018). However, a study conducted in Taiwan showed that lncRNA MALAT1 gene variants are not associated with the incidence, clinicopathological characteristics, or the 5-year survival rates of women with cervical cancer (Sun YH. et al., 2021).

A limitation of this review is that it only analyzed the relationship between MALAT1 and related respiratory diseases in terms of active or passive regulation, and did not investigate the role of MALAT1 in pathogenesis. This warrants further study. Whether MALAT1-regulated diseases can be treated by regulating MALAT1 directly is a potential avenue for future research.

This review of the empirical and clinical evidence describes the potential mechanisms by which MALAT1 regulates respiratory disease and suggests that it could serve as a therapeutic target in the development of effective novel drug treatments for respiratory disease.
