# Supplementary material for: Mechanistic studies of MALAT1 in respiratory diseases
Source: Front Mol Biosci. 2022 Nov 7;9:1031861. doi: 10.3389/fmolb.2022.1031861 (PMC9676952; doi:10.3389/fmolb.2022.1031861)
Supplement: Supplementary file 1 [file Table2.DOCX]

**Mechanistic studies of MALAT1 in respiratory diseases highlights**

- MALAT1 is a lncRNA that targets expression of multiple miRs via sponge adsorption
- MALATI induces various signalling pathways ex. PI3K/Akt, KEAP1/NRF2, Wnt/β-catenin
- MALAT1 either exacerbates or ameliorates existing respiratory diseases & conditions
- MALAT1 could serve as a predictive, prognostic, prophylactic, or therapeutic target
- Future research should aim to clarify the precise molecular mechanisms of MALAT1
